# Separating lexical-semantic access from other mnemonic processes in picture-name verification

**DOI:** 10.3389/fpsyg.2013.00706

**Published:** 2013-10-11

**Authors:** Jason F. Smith, Allen R. Braun, Gene E. Alexander, Kewei Chen, Barry Horwitz

**Affiliations:** ^1^Brain Imaging and Modeling Section, National Institute on Deafness and Other Communication Disorders, National Institutes of HealthBethesda, MD, USA; ^2^Language Section, National Institute on Deafness and Other Communication Disorders, National Institutes of HealthBethesda, MD, USA; ^3^Department of Psychology and Evelyn F. McKnight Brain Institute, University of ArizonaTucson, AZ, USA; ^4^Arizona Alzheimer's Disease ConsortiumPhoenix, AZ, USA; ^5^Department of Mathematics and Statistics, Arizona State UniversityPhoenix, AZ, USA; ^6^Banner Alzheimer's Institute and Positron Emission Tomography Center, Banner Good Samaritan Medical CenterPhoenix, AZ, USA

**Keywords:** fMRI, memory, crossmodal, language

## Abstract

We present a novel paradigm to identify shared and unique brain regions underlying non-semantic, non-phonological, abstract, audio-visual (AV) memory vs. naming using a longitudinal functional magnetic resonance imaging experiment. Participants were trained to associate novel AV stimulus pairs containing hidden linguistic content. Half of the stimulus pairs were distorted images of animals and sine-wave speech versions of the animal's name. Images and sounds were distorted in such a way as to make their linguistic content easily recognizable only after being made aware of its existence. Memory for the pairings was tested by presenting an AV pair and asking participants to verify if the two stimuli formed a learned pairing. After memory testing, the hidden linguistic content was revealed and participants were tested again on their recollection of the pairings in this linguistically informed state. Once informed, the AV verification task could be performed by naming the picture. There was substantial overlap between the regions involved in recognition of non-linguistic sensory memory and naming, suggesting a strong relation between them. Contrasts between sessions identified left angular gyrus and middle temporal gyrus as key additional players in the naming network. Left inferior frontal regions participated in both naming and non-linguistic AV memory suggesting the region is responsible for AV memory independent of phonological content contrary to previous proposals. Functional connectivity between angular gyrus and left inferior frontal gyrus and left middle temporal gyrus increased when performing the AV task as naming. The results are consistent with the hypothesis that, at the spatial resolution of fMRI, the regions that facilitate non-linguistic AV associations are a subset of those that facilitate naming though reorganized into distinct networks.

## Introduction

The ability to learn associations between stimuli in multiple sensory modalities is extremely important. Some of these associations can be based upon past experience with a bound multisensory object (e.g., seeing a tiger and hearing it growl at the same time). The visual and auditory stimuli are inherently related as they emanate from and are bound to the same object. Information from these individual episodes of memory can create an abstracted representation to guide future action. Hearing a similar growl in the bushes brings to mind the representation of the tiger with obvious survival advantages. In humans, production and comprehension of spoken language typically requires the association of an auditory representation and a semantic concept that often has a visual representation as well. Naming a picture of a tiger for example, is believed to require visual access to the semantic representation of “Tiger,” leading to selection of the appropriate lexical item associated with this concept (Levelt et al., 1991). However, these linguistic associations are not inherently related to the concepts in the world and are completely arbitrary (e.g., there are no affordances in the image of a tiger that suggests its auditory label should contain the phoneme /t/). The arbitrary word labels are not bound to the picture thereby forming a multisensory object but rather the multiple representations are associated together in memory.

Experimental data from non-human primates and other animals clearly indicate that arbitrary audio-visual (AV) or other cross-modal associations can be formed on the basis of behavioral relevance alone (Gibson and Maunsell, 1997; Fuster et al., 2000; Zhou and Fuster, 2000; Zhou et al., 2007; Kayser et al., 2008; Seki and Okanoya, 2008). For example, Fuster et al. (2000) trained rhesus monkeys to perform a delayed paired associate task matching pure high and low tones with red and green keys, respectively. Importantly, the auditory and visual stimuli were not presented simultaneously, thus the animals were not responding to a single multisensory object but rather two distinct stimuli associated with the same, also sensory, concept (a motor response and/or an expected reward) in memory. The findings of these studies indicate that cross-modal associations are at least in part represented by the activation of the different cortical areas involved in each sensory modality: inferior temporal cortex for visual stimuli (Miyashita, 1988), primary somatosensory cortex for tactile stimuli (Zhou and Fuster, 2000), primary auditory cortex and posterior auditory fields for auditory stimuli (Brosch et al., 2005; Kayser et al., 2008; Scheich et al., 2011). In addition these primary sensory areas, posterior and prefrontal association cortices also play a role in representing cross-modal associations (Fuster et al., 2000). The arbitrary associations are believed to be initially mediated via medial temporal cortex connecting the unimodal sensory cortices (McClelland et al., 1995; Miyashita et al., 1996, 1998; Frankland and Bontempi, 2005; Eichenbaum et al., 2007; Chen et al., 2013). Over time, these medial temporally mediated memory traces become independent of medial temporal structures and dependent upon posterior and prefrontal association cortices (Frankland and Bontempi, 2005). This view of arbitrary cross-modal long-term memory in non-human animals is analogous to the embodiment view of semantic memory in humans (c.f., Martin, 2001; Fuster, 2009; Binder and Desai, 2011). Neuroimaging evidence indicates that modality-specific cortical regions are involved in word processing and comprehension for items with specific sensory content. The involvement of this distributed network in word comprehension appears to be automatic, immediate, and essential (Bak and Hodges, 2004; Pulvermüller et al., 2005; Hoenig et al., 2008; Revill et al., 2008), though this view is not universal (c.f., Mahon and Caramazza, 2008). In the embodiment view then, part of understanding “Tiger” requires activation (though not necessarily conscious awareness) of imagery and sounds associated with tigers in addition to propositional knowledge.

Given the similarity of theories regarding the sensory cortex involvement in associating arbitrary AV memories in non-humans and the semantic representation of objects in humans, what then is the relationship between a concrete noun naming that object and any other arbitrary AV association? One plausible hypothesis is that, at least in early vocabulary acquisition, words, and referents are associated using this phylogenetically preexisting, cross-modal sensory association memory pathway (Wise et al., 2000; though see Cary, 2010; Sharon et al., 2011 for a contrasting view). In this view names are, at least initially, nothing more than arbitrary sensory AV associations. Evidence from infant language acquisition seems to support this view. Concrete nouns, in particular, exemplify this arbitrary cross-modal sensory association between a visual stimulus (an object) and an auditory stimulus (a spoken word). Concrete nouns are acquired earlier and used with greater proficiency by young children than abstract nouns (nouns without specific sensory referents) and words from other grammatical categories such as verbs where the sensory referent is more complex (Golinkoff et al., 1994; Tomasello et al., 1997). Infants readily learn novel object-sound associations after only a few presentations (Werker et al., 1998; Woodward and Hoyne, 1999) and may be biased to assume novel sounds including non-language sounds (e.g., whistles) are labels for novel objects (Gentner, 1982; Namy and Waxman, 1998). This preference for concrete nouns over abstract nouns and verbs has led several authors to conclude that concrete nouns are a privileged class in early language acquisition (Gentner, 1982; Kako, 2004; Gleitman et al., 2005). This privilege continues in healthy adults where the superior proficiency for concrete nouns remains (Kroll and Merves, 1986; Paivio, 1991) and in aphasic patients with left hemisphere surface lesions where concrete noun comprehension and retrieval are relatively preserved though the reverse deficit is rarely observed (Goodglass et al., 1969; Franklin, 1989; Breedin et al., 1994). Additionally infants as well as specific clinical populations are able to form arbitrary AV associations (names to objects) without understanding the “meaning” of the object itself (Kremin, 1986, 1988; Werker et al., 1998; Woodward and Hoyne, 1999; Funnell et al., 2006). The ability, shared with non-human animals, to arbitrarily associate sensory stimuli in memory may be a possible foundation for early language acquisition rather than the use of an evolutionarily novel language module unique to humans.

The goal of the current study is to investigate commonalities and differences between linguistic AV associations and arbitrary non-linguistic AV associations. By linguistic AV memory we mean all cognitive processes involved in accessing the auditory representation of the name of a visually presented object with the exception of overt production; in essence covert naming. We will, with mild abuse, use the term naming as short hand for this sequence of processes. By non-linguistic AV memory we mean a long-term memory for an association between an auditory and a visual stimulus without any meaning beyond the fact that the two items are related and where the individual items themselves have no a priori meaning, connection with other stimuli, or phonetic content. We hypothesize that non-linguistic AV associative memory is fundamentally related to naming such that the spatial distribution of the network underlying storage and recall of well learned AV associations is similar to the spatial pattern of the network underlying naming. We further hypothesize, however, that a distinct pattern of network interactions between these common regions as well as possibly additional lexical/semantic regions will emerge with linguistic mediation of the association.

There have been several studies investigating AV associations using overtly linguistic stimuli (e.g., written or spoken words; Calvert et al., 2000; Ojanen et al., 2005) and non-verbal semantically associated images and sounds (Beauchamp et al., 2004; Taylor et al., 2006; Thierry and Price, 2006; Hocking and Price, 2008). These studies identify a broad network of regions that support naming including regions in inferior temporal, inferior parietal and inferior frontal gyri and cortex surrounding the superior temporal sulcus (see Price et al., 2005 for a review). So called “control” tasks in these experiments often involve presentation of scrambled or distorted items that have no intrinsic or experimentally induced association. These experiments thus potentially confound lexical/semantic access with processes common to arbitrary associative memory. Abstract, non-linguistic AV associations have also been studied, though typically in the context of memory formation and consolidation (Tanabe et al., 2005; Smith et al., 2010; Pillai et al., 2013). These studies report a spatially similar set of brain regions involved in non-language AV memory though to our knowledge there has been no direct comparison between naming and non-linguistic AV associations in the same participants.

As a practical matter, direct experimental comparison of naming and non-linguistic AV processing is difficult. Ideally, a comparison would be made between for identical stimulus pairs to avoid extraneous differences due to low level stimulus features. However, access to the lexical-semantic system is automatic; when presented with a known item, participants will be unable to prevent semantic access and recall of the item name. Using images of real but unknown objects (e.g., Cornelissen et al., 2004) does not completely alleviate this concern as object affordances will suggest semantic categories and uses. Conversely, abstract images and sounds by definition contain no verbal content. Absent overtly linguistic mnemonic strategies (e.g., using a second level association of a word to an abstract sound or known object to an abstract image), participants will be unable to meaningfully associate these items via linguistic routes.

Here we describe a novel longitudinal neuroimaging paradigm designed to directly contrast non-linguistic associations from naming for identical AV stimulus pairs. Visual stimuli for the AV pairs were created by degrading photographs of common animals using low-pass filters and additional manipulations such that they were not recognizable as images of objects. However, the animal in the degraded image could be clearly and consistently identified after viewing the degraded image juxtaposed with original unfiltered images (Mooney, 1957; Ramachandran et al., 1988; Dolan et al., 1997; Kanwisher et al., 1997; Andrews and Schluppeck, 2003). Auditory stimuli were created by generating distorted sine wave speech versions of the animal's name which were also not immediately recognizable as linguistic sounds until heard in conjunction with the corresponding spoken name (Remez et al., 2001; Meyer et al., 2005). By training participants to associate these items in an uninformed state (i.e., unaware of the linguistic content), non-linguistic mediation of AV memory for these items can be examined. This condition is expected to be essentially similar to training a non-human primate to associate a light and a tone. Once participants are informed of and able to use the linguistic content hidden in the items, naming (i.e., linguistic AV memory) for the identical stimulus parings can be examined.

In the current study, we report comparisons between AV memory, as measured by a pair verification task, for these cross-modal items before and after participants were informed of the lexical nature of the pairings. To avoid uninteresting differences due to repetition effects or visual processing differences due to the presentation of unfiltered stimuli, we also examine memory for AV pairs that were created in an identical manner as the hidden linguistic items but based on arbitrarily paired abstract fractal patterns and non-words. These items can only be associated by “non-linguistic” memory as no meaningful associations exist with the fractal images and non-words. That is, knowledge of the un-degraded fractal would not bring to mind a semantic representation of that specific fractal which would in turn activate the specific non-word allowing the degraded fractal patterns to be “named.” Whatever means was used to associate these fractal-non-word items prior to the informed condition must still be used in the Informed session. However, to the extent that the participants hear the non-word in the informed condition, non-lexical, non-semantic linguistic processes (e.g., phoneme segmentation and processing, prosody, etc.) will also be activated by the stimuli and thus controlled for in the comparison between conditions. By comparing animal/name pairs in uninformed and informed states, regions directly involved in linguistic/semantic mediation distinct from non-lingusitic associative memory can be identified and their inter-regional interactions examined. Again, we hypothesized that the regions participating in the non-linguistic AV pair verification task would be a subset of those participating in the naming version of the task. We further hypothesized that the additional lexical/semantic regions would become functionally connected with the regions participating in the non-linguistic network.

## Experimental procedures

### Participants

Twenty-three, healthy, monolingual, right handed paid volunteers with normal hearing and normal or corrected to normal vision were initially recruited for the experiment and gave informed consent in accordance with the procedures of the National Institutes of Health. Participants had no reported history of drug abuse, mental illness, or head trauma, and were medically screened to exclude neurological, psychological, and cardiopulmonary disorders, and the use of medications acting on the vascular system. Participants reported no significant study or knowledge of foreign language, music, or visual arts. Of these 23 participants, six were excluded for failing to learn the task to 90% accuracy in the initial training session, two were excluded for failing to return for all follow-up sessions, two were excluded for poor performance inside the scanner environment, and one was excluded for excessive motion during scanning. Data from only the 12 remaining Participants (7 females, mean ± std. age = 25.75 ± 3.44) were analyzed further.

Participants' visual acuity was tested in the scanner environment using a projected Snellen chart and confirmed to approximate 20/20 vision. Audiological testing was performed on six of these subjects and confirmed to be within normal limits. Normal hearing was confirmed in the remaining six subjects via self report. Right handedness was confirmed using the Edinburgh Handedness Inventory (Oldfield, 1971). Participants self-reported compliance with a request to abstain from alcohol, tobacco, caffeine, prescription, and non-prescription medications for at least 4 h prior to all experimental sessions.

### Stimuli

**Figure 1:**
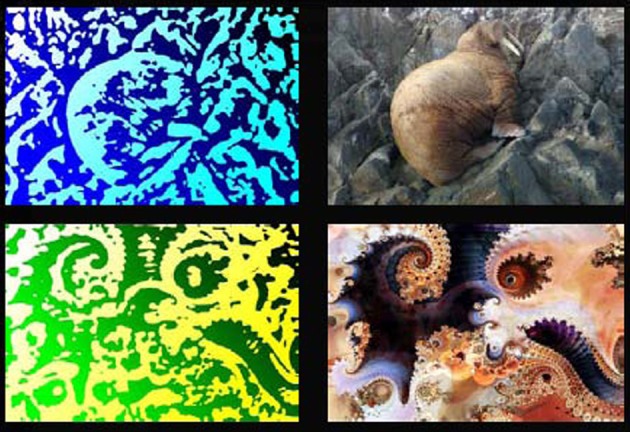
**Example experimental visual stimuli**. Two example visual stimuli from the experiment are shown. The images to the **left** are actual stimuli used in the experiment. The images to the **right** are the unfiltered images from which the experimental stimuli were created. All scanning trials used the filtered (left) stimuli. Fractal images were used with permission from www.fractalrecursions.com.

Stimuli in the experiment consisted of 18 pairs of visual and auditory stimuli. The visual stimuli were generated using low pass filters implemented in Photoshop (Adobe Inc.) from nine photographs of common animals and nine non-nameable fractal images (http://www.fractal-recursions.com/). All images were converted to gray-scale and the filters “photocopy” and “stamp” were applied in succession. Two-tone color was then added back into the images to make them more distinguishable. The resulting images were not immediately identifiable and participants were told that no meaningful information was contained in the images (see Figure 1). Auditory stimuli were created using a modified sine wave speech conversion process, implemented in Matlab (MathWorks Inc.) and based on a LPC filter (http://labrosa.ee.columbia.edu/matlab/sws), from nine spoken animal names and nine non-words matched to the animal names for length in phonemes, syllable number, and mean phoneme-to-phoneme transition frequency. Speech sounds (both words and non-words) were generated using a text-to-speech program with multiple synthetic voices (http://www.naturalvoices.att.com/demos). The three prominent formants of each spoken item were modeled and shifted in frequency such that the mean of each of the three formants varied around the three notes of major chords. Harmonics were added to the notes resulting in a modulated chord-like sound. The resulting sounds were not immediately identifiable as speech and participants were told that though the stimuli had some low level features of speech they were not identifiable speech sounds (see Supplementary material for examples). Participants were told that the experiment was to contrast stimulus complexity in the absence of language and that all stimuli were non-nameable. Post-experiment debriefing questionnaires indicated that two participants identified one animal image and one participant identified one animal name prior to being informed of the linguistic content. Trials containing these items were excluded from all analyses for these participants. The remaining participants indicated having no knowledge of the linguistic nature of the stimuli prior to the final session.

### Task

On the first day of the experiment, participants were seen for a medical examination, audiological testing if possible, and handedness testing. Participants were shown 30 random pairings of the visual stimuli and asked select the most complex of each pair. They then listened to 30 random pairings of auditory items and asked for the same rating. The purpose of these ratings was to exclude participants who were able to identify the linguistic nature of the stimuli and to instill in them the belief that the pairings would be based on non-linguistic features. Participants returned a minimum of one and a maximum of three days later and were trained to associate the 18 pairs. During training, participants were presented with the correct pairs three times each and then performed a pair verification task with feedback on their response accuracy. In the pair verification task a picture was presented followed by a brief delay then a sound. Participants indicated if the sound was or was not the correct pair to the image. This presentation-test cycle was iterated a minimum of four times and for a maximum of 1.5 h until the participant achieved a 90% accuracy level on the test. After achieving the accuracy criterion, participants were placed in the scanner and given one more presentation of the correct pairings with the scanner running. Imaging data were then collected while participants performed 72 delayed picture-sound verification trials over three scanning runs. Each image was presented four times over the three runs, twice in a correct pairing and twice in an incorrect pairing and never twice correctly in the same run. Each sound was also presented four times with the same criteria. Participants returned 2 weeks later and were tested on their recall of the pairs outside the scanner. They were then retrained, as before, to again achieve 90% accuracy. Participants then returned exactly 28 days after the initial training/scanning session and again performed 72 pair verification trials over three scanning runs.

After this session the linguistic nature of the stimuli were revealed. Participants were told of the true nature of the stimuli and the linguistic nature of half of the pairings. Participants were shown each degraded and non-degraded visual stimulus together and told to examine closely the relation between the pictures. They were then presented with sine wave speech nursery rhymes to acquaint them with degraded speech followed by the filtered and unfiltered experimental auditory stimuli. Participants were asked to try to hear the unfiltered sounds in the filtered versions. The visual and auditory stimuli were never paired during this session though subjects were told that the correct pair of an animal image was the animal name. After revealing the stimuli, participants were again scanned while performing the same pair verification task with the degraded stimuli. Debriefing tests were used to determine if participants could recognize the degraded stimuli and if any stimuli were recognized prior to the final session.

In each scanned trial, an image was presented for 2 s followed by a 5–8 s delay (mean 6.5 s) consisting of a white fixation cross on a black screen, followed by a sound with durations around 1 s. The sound was the correct pair of the image in 50% of the trials. Subjects responded via button press, using the left hand for a correct pairing and the right hand for an incorrect pairing, during the 4–18 s inter-trial interval. Auditory foils were taken from the targets of other images; no novel images or sounds were presented. Ordering of correct and incorrect pairs and animal or fractal pairs was controlled by M-sequences (Buracas and Boynton, 2002) to ensure the orderings were unpredictable. The trial ISI and ITI timings were selected to minimize the correlation between the expected hemodynamic response regressors and maximize the regression tolerance to better observe the evolution of the task related BOLD signal over time (Smith et al., 2010).

### Imaging

Functional imaging data were collected with a 3 Tesla GE system (General Electric, Milwaukee, WI), using the ASSET EPI parallel imaging sequence (acceleration factor of 2) and an eight channel head coil. Thirty-two T2^*^-weighted EPI axial images (*TR* = 2000 ms, *TE* = 30 ms) were collected in an interleaved order with a 2.6 mm slice thickness, 1.2 mm slice gap, and a 2.5 mm by 2.5 mm within plane resolution (96 by 96 Matrix, 240 mm FOV). Each scanning run consisted of 232 volumes and the first four were excluded to avoid saturation effects. Two structural images were acquired at the first scanning session. Both structural images were T1 weighted SPGR images. One image was collected with the same voxel size at the same slices as the functional images (*TE* = 2.4 ms, Flip angle 70°). A second, full brain image was also acquired at higher resolution, optimized to maintain short acquisition time (220 mm FOV, 224 by 224 Matrix, 1 mm by 1 mm in plane resolution, 1.3 mm slice thickness, 128 slices, TE 2.7 ms, Flip angle 12°, Prep time 450 ms).

### Data analysis

Analysis of the functional imaging data was performed using the SPM2 software package (http://www.fil.ion.ucl.ac.uk/spm) and additional routines implemented in Matlab (MathWorks). Images were corrected for slice acquisition time effects, realigned across all sessions and runs using a six parameter, rigid body transformation. The low resolution T1 anatomical image was similarly coregistered to the mean EPI image and the high resolution T1 image was coregistered to this low resolution T1 image. The high resolution anatomical image was segmented into separate gray matter, white matter and CSF components and the gray matter image was non-linearly warped to a gray matter template image in the MNI atlas space. The resulting normalization parameters were applied to the coregistered EPI images which were then resliced to 2.5 mm isotropic voxels using 7th degree B-spline interpolation. EPI images were smoothed with an 8 mm FWHM Gaussian kernel.

Individual subject analyses were performed using a restricted maximum likelihood multiple linear regression. The canonical mixture of two beta functions model of the hemodynamic response was used to separately model the visual cue, delay period, and auditory response components of trials of each type. All responses were relative to the implicit common baseline. This approach to modeling multiple parts of single trials and delay period activation in particular has been successfully performed in several publications (Zarahn et al., 1999; Postle et al., 2000; Zarahn, 2000; Barde and Thompson-Schill, 2002; Pessoa et al., 2002; Druzgal and D'Esposito, 2003; Curtis et al., 2004; Gazzaley et al., 2004; Ranganath et al., 2004; Rissman et al., 2004; Postle, 2006; Smith et al., 2010). Correct match and non-match auditory response components were modeled separately and additional regressors not of interest were used to model incorrect trials, subject motion, and image run means. To avoid considering guesses as correct, trials with reaction times 2.5 standard deviations beyond the run mean or greater than 3 s, and individual runs where the 95% confidence interval on the within run *d*'-value crossed 0 were excluded from image analyses.

Mixed effects analyses across participants were performed via a two step summary statistic procedure used in fMRI data analysis (Holmes and Friston, 1998; Beckmann et al., 2003; Friston et al., 2005; Mumford and Nichols, 2006; Mumford and Poldrack, 2007; Poldrack et al., 2011). Contrast images from the individual participant analyses were used as the basis for testing the generalizability of the individual participant effects. Separate ANOVAs, corrected for non-sphericity and subject specific effects, were used to test for session differences in BOLD responses during each trial subcomponent (visual cue, delay, and response) of correct trials.

The research question of interest centered on the difference in BOLD response to animal-name pairs before and after being informed of their linguistic nature. Therefore, subsequent analyses focused on session differences for correct animal/name trials. Fractal/non-word served as high-level control trials (c.f., Price et al., 2005). The BOLD response to fractal/non-word items should be identical across sessions since knowledge of the unfiltered fractal image and unfiltered non-words provides no assistance in identifying correct pairings. Therefore, any session difference for fractal/non-word trials would be due to the extraneous effects of increased exposure, additional trial repetitions, fatigue, and recall of the unfiltered images and sounds. To avoid these uninteresting session differences, analyses of animal/name BOLD responses were masked to exclude any regions showing session differences in fractal/non-word trials at a *p* < 0.05 uncorrected level. All reported results are corrected for multiple comparisons to *p* < 0.05 at the cluster level. Cluster level inferences are more sensitive than voxel level inferences while still controlling for multiple comparisons though the accurate localization of effects is reduced (Friston et al., 1996).

Univariate analysis identified a region of interest in the vicinity of the angular gyrus (AG). To better interpret the involvement of this region, functional connectivity with this region was examined using a modified psychophysical interaction analysis (PPI) analysis (Friston et al., 1997; Gitelman et al., 2003). PPI models the task induced changes in the regression slope between two regions and is a well established method (see O'Reilly et al., 2012 for a recent review). Specifically, PPI was used to identify regions more connected with the identified AG region in the Informed than in the Uninformed condition during the delay period. To isolate the delay period for subsequent connectivity analysis, regression coefficients were estimated for each individual trial using a general linear model to estimate the hemodynamic response magnitude on a per-trial basis (Gazzaley et al., 2004; Rissman et al., 2004). Within each trial, the cue, delay, and target components were modeled separately. The full model also included estimated motion parameters, individual run means, and a band-pass filter. The delay period regression coefficients for each trial were extracted from this model and further analyzed using PPI. This modified PPI analysis is logically similar to a PPI performed on PET data and allows connectivity to be estimated for portions of long, complex trials where the connectivity is likely to change within a trial (Rissman et al., 2004).

For each comparison discussed below, the delay regression coefficient series for each condition was centered such that the series for each voxel in each run in that condition had a mean of zero and unit variance. This explicitly removes the main effects of run, session, and condition from the data. The seed region for the PPI analysis was created by taking the regression coefficient series from a 6 mm cube centered on the local maximum in the AG and calculating the first eigenvariate from this cube. Target voxels for the PPI included only those voxels that had reliably greater BOLD signal during the delay portion of the task than baseline in the Uninformed condition. This restricts the analysis to only voxels potentially involved in the task during the delay period.

## Results

Comparisons of the sessions immediately after learning and 28 days later in the uninformed state were reported elsewhere (Smith et al., 2010). These comparisons showed evidence of consolidation of the AV associations into long-term memory. Below we focus on the uniformed (before participants were aware of the linguistic content of the stimuli) and informed (after participants were aware of the linguistic content) sessions collected during the 28 day follow-up visit.

### Behavior

Over all trials, participants responded accurately in both sessions (mean ± std. Uninformed = 74.31% ± 7.29; Informed = 78.12% ± 8.81). Accuracy during the Informed session did not reliably differ from Uniformed session when analyzed using a parametric (*t*_11_ = 1.48, *p* = 0.17) or a non-parametric (Wilcoxon *T* = 13.5, *p* = 0.17) test. Participant sensitivity (*d*') was also at acceptable levels (mean ± std. Uninformed = 1.52 ± 0.55; Informed = 1.83 ± 0.51). Sensitivity reliably differed between the sessions when analyzed using a parametric test (*t*_11_ = 2.33, *p* = 0.04), but did not reliably differ when analyzed using a non-parametric test (Wilcoxon *T* = 18, *p* = 0.10).

Comparing responses to target present animal-name trials and target present fractal-nonword trials within the Uninformed session, accuracy levels for the two trial types were similar (mean ± std. Uninformed target present animal-name = 74.07 ± 13.04%, Uninformed target present fractal-nonword = 75.46 ± 8.69%) and did not reliably differ (Wilcoxon *T* = 25, *p* = 0.87). Within the Informed session, accuracy levels for the two trial types had a larger difference (mean ± std. Informed target present animal-name = 83.33% ± 12.08%, Informed target present fractal-nonword = 76.38 ± 15.56%) and reliably differed (*t*_11_ = 2.38, *p* = 0.04; Wilcoxon *T* = 48, *p* = 0.043). There was also a difference between the sessions for target present animal-name trials with greater performance in the Informed session (*t*_11_ = 2.59, *p* = 0.025; Wilcoxon *T* = 48, *p* = 0.041) but not for target present fractal-nonword trials (*t*_11_ = 0.26, *p* = 0.82; Wilcoxon *T* = 36.5, *p* = 0.82).

When accuracy was collapsed across target present and target absent trials, the pattern of results was numerically similar as when only target present trials were considered (mean ± std. Uninformed animal-name = 75.93 ± 10.55%, Uninformed fractal-nonword = 75.46 ± 7.66%, Informed animal-name = 81.25 ± 8.30%, Informed fractal-nonword = 76.85% ± 10.55%). However, despite the similarity, none of the differences were reliable when analyzed using parametric or non-parametric tests (all *p* > 0.1).

After scanning, participants were tested on their ability to identify the animals within the degraded images in a confrontation naming test. When presented with a degraded animal picture, participants were able to produce the animal name with considerable accuracy (mean ± std. 96.3% ± 5.4). A similar procedure was used to test participants' ability identify the degraded animal names when presented in isolation (mean ± std. 96.3% ± 5.4). Participants were much less able to identify the degraded names in isolation (mean ± std. 46.30% ± 14.86). However, participants could perform a verification task where a written animal name was followed by an experimental sound and subjects indicated whether a match occurred (mean ± std. 82.0% ± 8.8). Participants were thus able to recognize the animal names when cued as in the AV pair verification task. The ability of participants to accurately name the degraded animal images after exposure to the undegraded forms, accurately identify the degraded auditory animal names when cued, and the increased accuracy in the informed condition for animal-name items but not fractal-nonword items strongly suggests participants used a lexical/semantic strategy to recall the animal pairs in the Informed session.

### Imaging

**Figure 2:**
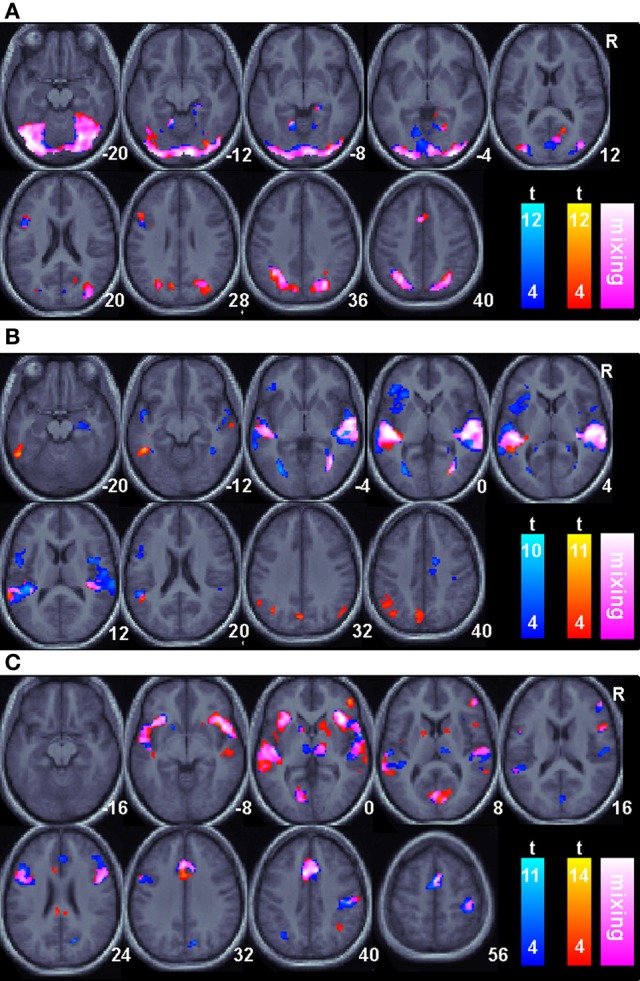
**Task related BOLD increases for correct trials relative to an arbitrary resting baseline for all sessions**. Only areas surviving a *p* < 0.05(corrected) cluster level threshold are shown on the mean normalized anatomical image from all twelve subjects. The Non-linguistic session (N) is shown in a blue-cyan scale and the Informed, linguistic session (L) is shown in red-yellow scale. Overlapping areas are indicated by color mixing according to the color scale as shown in the legend. **(A)**. The Visual cue portion of the task. **(B)**. The Delay period portion of the task. **(C)**. The Auditory target portion of the task.

Regions of BOLD signal increase relative to fixation for correct animal-name trials are shown in Figure 2 and Table 1. The figure shows areas unique to each session as well as areas common across sessions. Common areas represent regions where both conditions exceed the threshold and thus are logically equivalent to the minimal t statistic used in conjunction analysis with the conjunction null (Nichols et al., 2005). However, regions that appear in only a single color scale so not indicate statistically reliable differences across sessions; the figure merely plots the within session results on the same background to facilitate comparisons. During the visual cue period, shown in Figure 2A, overlapping or nearly overlapping BOLD signal increases are seen posterior regions, as well as the right hippocampus, anterior cingulate cortex extending into supplementary motor area and left pars opercularis. During the delay period, shown in Figure 2B, substantial overlap in BOLD signal increases occurred in the superior temporal sulcus extending into both the superior and middle temporal gyri bilaterally. BOLD increases unique to the Informed session occurred in the bilateral angular gyri, precuneus, and left inferior temporal sulcus. During the auditory target period, shown in Figure 2C for match trials only, substantial overlap in BOLD related signal increases occurred in the bilateral insula, bilateral superior temporal gyrus, anterior cingulate extending into supplementary motor area, bilateral inferior frontal gyri including pars triangularus and pars opercularis, primary visual cortex, right ventral thalamus, and the right motor cortex in the vicinity of the hand area. BOLD increases for the Uninformed session alone occurred in the precuneus and left intraparietal sulcus.

**Table 1:** **Regions of BOLD signal increase relative to fixation for correct animal-name trials**.

**Uninformed session animal/ name > fixation**			**Informed session animal/ name > fixation**		
**MNI coordinate [*x, y, z*]**	***k_E_***	***p*_Corrected_**	**MNI coordinate [*x, y, z*]**	***k_E_***	***p*_Corrected_**
**VISUAL CUE**					
[−12, −50, −10]	4774	<0.001	[30, −92, 0]	4597	<0.001
[−25, −62, 40]	478	<0.001	[−35, −58, 38]	651	<0.001
[−2, 12, 45]	88	0.001	[−50, 20, 28]	117	<0.001
[8, −75, 48]	389	<0.001	[−15, −75, 2]	109	0.001
[−45, 10, 20]	120	<0.001	[0, 5, 52]	110	0.001
			[8, −45, 0]	63	0.014
**DELAY**					
[55, −22, 8]	1620	<0.001	[32, −25, 5]	916	<0.001
[−40, −35, 10]	1862	<0.001	[−45, −25, 0]	733	<0.001
[−30, −70, −2]	193	<0.001	[−48, −45, −12]	133	<0.001
[−18, −2, 60]	248	<0.001	[28, −72, 0]	80	0.002
[32, −65, −2]	213	<0.001	[−38, −65, 35]	228	<0.001
[15, −2, 42]	132	<0.001	[−2, −72, 32]	122	<0.001
[35, −18, −22]	56	0.007	[−35, −32, 52]	98	0.001
[42, −18, 45]	53	0.010	[50, −68, 28]	60	0.011
[−15, −42, 10]	53	0.010			
**AUDITORY TARGET**					
[52, −15, 45]	552	<0.001	[40, 25, −5]	1425	<0.001
[−5, 15, 42]	885	<0.001	[−2, 25, 30]	689	<0.001
[12, −72, 30]	75	0.003	[−58, −15, 0]	1187	<0.001
[38, 20, 2]	954	<0.001	[−2, −72, 8]	309	<0.001
[−42, 10, 20]	366	<0.001	[−5, −32, 22]	48	0.024
[−45, −18, −2]	958	<0.001	[52, −18, 48]	354	<0.001
[50, 8, 25]	331	<0.001	[40, −58, −35]	74	0.002
[−15, −52, −30]	126	<0.001	[18, 18, −5]	140	<0.001
[10, −20, 5]	207	<0.001	[42, −55, 48]	64	0.005
[−18, 12, −5]	52	0.020	[−22, −58, 48]	135	<0.001
[2, −80, 15]	255	<0.001	[−48, 10, 22]	199	<0.001
[−35, −65, 42]	46	0.035	[−15, 2, 8]	45	0.032
			[15, −12, 0]	58	0.009

Peak location (in MNI coordinates), cluster volume (in voxel count), and correct p values for clusters identified as having greater BOLD response during animal-name trials versus baseline for each trial sub-component in the Uninformed (left side columns) and Informed (right side columns) sessions.

Regional differences between the Informed and Uniformed sessions were identified by selecting regions with reliably greater BOLD response between sessions for animal-name trials (*p* < 0.05 corrected for multiple comparisons at the cluster level) while excluding regions with greater BOLD response between sessions for fractal-nonword trials (*p* < 0.05 uncorrected at the voxel level). This exclusive masking removes any condition independent repetition effects such as effects of seeing the undegraded images.

**Figure 3:**
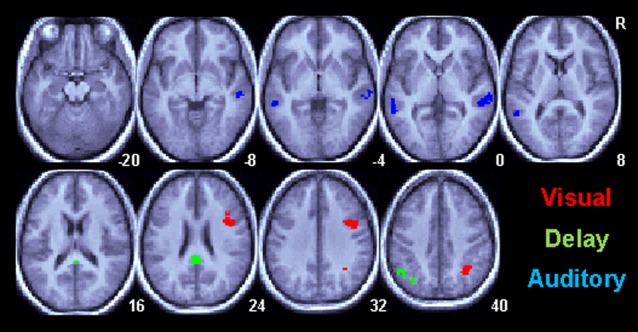
**Task related BOLD increases for correct trials in the Informed/Linguistic session relative to the non-linguistic session for all subcomponents of the task**. Only areas surviving a *p* < 0.05(corrected) cluster level threshold are shown on the mean normalized anatomical image from all twelve subjects. The Visual cue (V) period is shown in red, the Delay (D) period is green and the Auditory (A) target period is shown in red.

Regional differences between the Informed and Uninformed sessions are shown in Figure 3 and Table 2. Session differences during the visual cue period of the task are shown in Figure 3 in red. During the visual cue period, knowledge of the linguistic content in the Informed session increased the magnitude of the BOLD response relative to the Uninformed session in the right ventral intraparietal sulcus in a region corresponding to HIP1(Caspers et al., 2006) and in the right pars opercularis (Brodmann area 44; BA44). Session differences during the delay period of the task are shown in Figure 3 in green. During the delay period, knowledge of the linguistic content in the Informed session increased the magnitude of the BOLD response relative to the Uninformed session in the left AG and the posterior cingulate cortex (PCC). Session differences during the auditory target period of the task are shown in Figure 3 in blue. Again, these results are for match trials only. During the auditory target period, knowledge of the linguistic content in the Informed session increased the magnitude of the BOLD response relative to the Uninformed session in the bilateral superior temporal sulci extending down into the middle temporal gyri.

Table 2**Regional differences in BOLD signal between the Informed and Uninformed sessions**.

**Table:** 

	**MNI coordinate [*x, y, z*]**	***k_E_***	***p*_corrected_**
**Informed animal/name > Uninformed animal/name masked by Informed fractal/non-words > Uninformed fractal/non-words (exclusive mask, *p* < 0.05 uncorrected)**			
Visual cue	[32, −55, 38]	97	0.029
	[38, 8, 28]	183	0.001
Delay	[−2, −40, 25]	99	0.015
	[−48, −58, 42]	95	0.018
Auditory target	[65, −30 0]	129	0.008
	[−58, −38, 0]	113	0.015

**Table:** 

**Uninformed animal/name > Informed animal/name masked by Uninformed fractal/non-words > Informed fractal/non-words (exclusive mask, *p* < 0.05 uncorrected)**			
Delay	[55, −10, 18]	103	0.012

Peak location (in MNI coordinates), cluster volume (in voxel count), and correct p values for clusters identified as having greater BOLD response during animal-name trials in the Informed session versus the Uninformed session (top) and greater BOLD response during animal-name trials in the Uninformed session versus the Informed session (bottom).

There were no regions with greater BOLD response to the animal-name trials in the Uninformed session than in the Informed sessions during the visual cue and auditory target portions of the task. During the delay portion of the task a single region in the right rolandic operculum [55, −10, 18] was identified as having greater BOLD signal in the Uninformed than then Informed task for animal-name trials (*k*_E_ = 103, *p* = 0.012_corrected_).

### Connectivity

**Figure 4:**
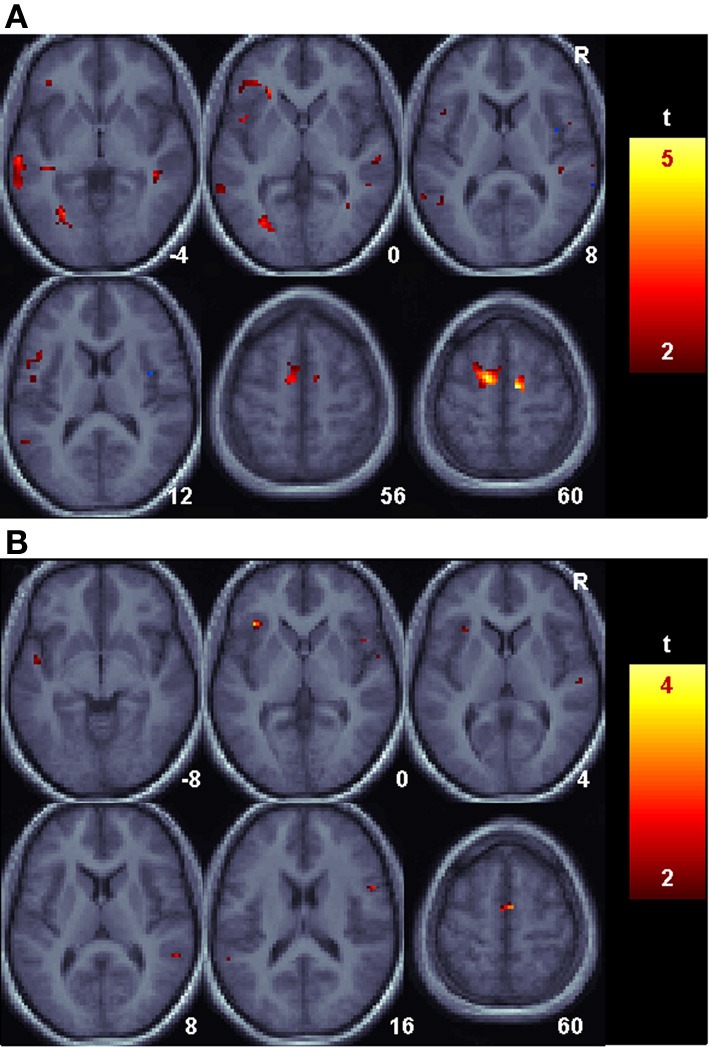
**Language related changes in functional connectivity with the angular gyrus**. Shown are areas with reliably greater BOLD signal relative to baseline for the Uninformed, non-linguistic session that nonetheless had reliably greater functional connectivity with the angular gyrus during the Informed, linguistic session. **(A)**. The Delay period portion of the task. **(B)**. The Auditory target portion of the task.

A voxel [−48, −58, 42] in the vicinity of the AG was selected from the peak of the univariate analysis during the delay period. This voxel has a 60% probability of being in the AG (PGa) and a 40% probability of lying in area PFm; the transition between the angular and supramarginal gyri (Caspers et al., 2006, 2008). Using a paired *t*-test, the regression slope for correct animal/name trials was compared between the Informed and Uninformed sessions to identify regions more connected to the AG during the Informed condition. The search was restricted to only those regions with reliably increased BOLD signal during the Uninformed sessions. The results are shown in Figure 4A for the delay portion of the task. During the delay period, several foci active in the Uninformed session in the left middle and superior temporal gyri, left IFG, and bilateral supplementary motor cortex were more connected to the left AG in the Informed session than in the Uninformed session. Similar increases in connectivity, though smaller is spatial extent, were seen during the auditory target portion of the task (Figure 4B).

## Discussion

To our knowledge, the current study is the first to directly compare and contrast regional BOLD responses associated with well learned non-linguistic AV associations and naming for identical stimuli. This direct comparison was used to distinguish regions involved specifically in lexical/semantic access from those involved in non-linguistic long-term associate memory. Substantial overlapping BOLD signal increases relative to the simple baseline occurred bilaterally in primary visual and visual association areas, medial temporal regions, middle and superior temporal gyri, anterior cingulate cortex, insula, and the inferior frontal gyrus (IFG). This generic overlap between the Informed (naming) and Uninformed (non-linguistic) sessions is consistent with the hypothesis that a similar set of regions participate in recall of AV associate memory regardless of the linguistic content of the stimuli. Further, the spatial pattern of overlapping regions is qualitatively similar to that seen in studies of reading and naming as well as studies of non-linguistic AV paired associate tasks in humans (c.f., Price et al., 2005; Tanabe et al., 2005; Smith et al., 2010).

Despite the strong global overlap in the BOLD increases seen in both sessions, several regions did show greater BOLD signal in the Informed than Uninformed sessions. Thus, these regions are specifically implicated in lexical/semantic processing. During the visual cue portion of the task, BOLD increases in the Informed relative to the Uninformed session were located in right intraparietal sulcus in the vicinity of HIP1 and the right IFG most likely in BA44. During the delay portion of the task, BOLD increases in the Informed relative to the Uninformed session were located in the left inferior parietal lobule, with the majority of the cluster in the left angular gyrus (PGa), and in the PCC. During the auditory target portion of the task, BOLD increases in the Informed relative to the Uninformed session were located bilaterally on the middle temporal gyrus.

Of these five regions exhibiting greater BOLD signal in the Informed than Uninformed sessions, the left AG and middle temporal gyri have frequently been implicated in lexical-semantic memory. The left AG in the inferior parietal lobule is considerably larger and more complex in its sulcal structure in humans than its homolog in non-human higher primates and may not fully exist in lower primates (Hyvarinen, 1982; Choi et al., 2006). It has been strongly implicated in some studies of semantic processing (Hart and Gordon, 1990; Vandenberghe et al., 1996; Price et al., 1997; Price, 2000; Vigneau et al., 2006; Ben Shalom and Poeppel, 2008) and showed the densest concentration of activation foci in a recent meta-analysis of semantic memory (Binder et al., 2009). Strong structural connections exist between the AG and the middle temporal gyrus as well as other linguistically related temporal areas via the middle longitudinal fasciculus and the posterior segment of the arcuate fasciculus and between the AG and Broca's area via the third branch of the superior longitudinal fasciculus (Catani et al., 2005; Frey et al., 2008; Kelly et al., 2010). Its proximity to multiple unimodal areas makes it a likely candidate for a supramodal integration area (Binder et al., 2009). While several studies have suggested that the AG is involved in semantic processing only at the sentence or discourse level (Dronkers et al., 2004; Xu et al., 2005; Vigneau et al., 2006), the current results are consistent with the hypothesis that the AG is a prominent part of the human lexical/semantic network at the single word level.

The middle temporal gyrus has also been implicated as part of the human lexical/semantic network in the Binder et al. (2009) meta-analysis as well as other reviews of neuroimaging data (Cabeza and Nyberg, 2000; Indefrey and Levelt, 2004; Price et al., 2005; Vigneau et al., 2006). This region has been identified most closely with lexical access or comprehension at the single word level (Hart and Gordon, 1990; Kaan and Swaab, 2002; Dronkers et al., 2004; Indefrey and Levelt, 2004). The greater relative BOLD signal in the Informed than in the Uninformed session demonstrated in the current study is consistent with the hypothesis that participants indeed used a linguistic strategy during the Informed session.

A commonly identified linguistic region notably absent from the between session comparisons of the current study is the left IFG. In studies of word retrieval, the left IFG has been associated with activation of and selection among entries in the mental lexicon (Petersen et al., 1989; Wagner et al., 2001; Sharp et al., 2005). However, the homolog of this region has specifically been shown to be involved in non-linguistic AV memory in monkeys (Fuster et al., 2000) suggesting the region is likely responsible for activation and selection of any AV association. This is consistent with the results of the current study. Here, the left IFG exhibited greater BOLD signal than baseline in both the Uninformed and Informed sessions (see Figure 2). The IFG exhibited greater BOLD signal than baseline for both the delay and target portions of the task for the Uninformed session while the IFG exhibited greater BOLD signal than baseline for only the target portion of the task in the Informed session. Previous studies have suggested that anterior portions of IFG (BA 45/47) are involved in controlled processing of semantic information while posterior IFG (BA 44) is involved in controlled processing of phonological information (Poldrack et al., 1999; Wagner et al., 2001). We observed BOLD increases posterior IFG in both sessions though phonological information was present in only the informed session. Thus, the current study does not provide evidence to support specialization for explicitly phonological information in posterior IFG.

We hypothesized that in the Informed condition, lexical/semantic (naming) regions would become functionally connected with regions participating in the non-linguistic network that was active during the Uninformed condition. We found evidence consistent with this hypothesis with several regions showing greater connectivity with the AG in the Informed than in the Uninformed session. Importantly, all of the regions examined for changes in connectivity were selected specifically for their task related activity in the Uninformed session. Thus, these regions were part of the network for non-linguistic AV associations. The posterior IFG region identified in the PPI analysis as more connected to the AG during linguistic conditions had increased BOLD signal relative to baseline in both sessions. Thus, while the activity of this region was similar in both session and conditions, the connectivity was not. These results are again consistent with the hypothesis that overt linguistic content in AV stimuli alters connectivity in non-linguistic AV networks rather than altering them spatially. The PPI result is inconsistent with theories that posit a strong specialization for phonological processing in posterior IFG (e.g., Poldrack et al., 1999; Wagner et al., 2001) and suggest a rather more nuanced hypothesis that the posterior IFG can become part of a circuit specializing in lexical or phonological processing, but that it can also become part of a non-linguistic AV circuit. This result is in line with an earlier finding by Bokde et al. (2001) that showed that fMRI activation was not sufficient to show a distinction between different parts of the IFG during phonological and semantic processing, but that functional connectivity with posterior brain areas could demonstrate such differences. Unfortunately, the spatial resolution of fMRI used here cannot rule out the possibility that subregions within the left IFG focus are specialized for phonology while others are not. In addition, statistical power of the current study is limited and cannot rule out differential BOLD signal magnitude in the left IFG between the sessions that was small in magnitude relative to the identified regional differences.

The remaining regions with greater BOLD signal in the Informed than Uninformed session have not typically been associated with lexical/semantic processing. The ventral intraparietal sulcus (HIP1) has been implicated in a variety of spatial representation, visual search, and visual working memory tasks (Coull and Frith, 1998; de Jong et al., 2001; Cohen and Andersen, 2002; Xu and Chun, 2005, 2009; Egner et al., 2008). The region was also observed in a previous study using similar degraded stimuli and thus may be involved in resolving the object in the degraded image (Dolan et al., 1997).

Though the left hemisphere homolog is more associated with phonological processing, the right IFG is reliably active during picture naming, pseudoword reading, as well as other linguistic tasks with overt or covert production (Indefrey and Levelt, 2004) or variations in task difficulty (Postman-Caucheteux et al., 2010). The right IFG, though more active in the Informed than Uninformed session, was active in both sessions but was not more functionally connected to the AG in the Informed session. Thus while the region did exhibit greater BOLD signal, we did not find evidence it was integrated into the lexical/semantic network *per se*. Finally the BOLD signal in PCC did exceed baseline levels during the Informed session but did not reliably exceed baseline levels during the Uninformed session. This suggests that similar to the AG, the PCC is possibly involved in lexical/semantic memory. However, we did not find evidence that the PPC was more functionally connected to the AG in the Informed session. A recent meta-analysis of fMRI studies of semantic memory consistently identified PCC as part of the human semantic network (Binder et al., 2009). They hypothesized that given the strong anatomical links between the PCC and the hippocampal complex, this region may serve as the interface between episodic memory and semantic memory. Several studies of episodic memory implicate the PPC in recognition of pictures and sounds (Wiggs et al., 1999; Shannon and Buckner, 2004) as well as other stimuli. BOLD signal in PCC during memory retrieval is related to depth of processing during encoding as well as detailed re-experiencing of the remembered item (Shannon and Buckner, 2004; Wheeler and Buckner, 2004). Thus, we believe the fact that participants last experienced the experimental stimuli 14 days prior in the Uninformed session but mere minutes prior in the Informed session as well as the increased depth of encoding afforded by the linguistic items accounts for the increased PCC signal.

A single region in the rolandic operculum was identified with a larger response to animal-name trials in the Uninformed session than in the Informed session during the delay period of the task. This region is near the somatosensory representation of the tongue and larynx (Pardo et al., 1997) and has been implicated in non-lyrical singing, processing pleasant music, and attending to the intonational contours and melody of speech (Riecker et al., 2000; Meyer et al., 2004; Koelsch et al., 2006). This again suggests participants switched from the more difficult non-linguistic auditory processing to linguistic processing once the nature of the animal-name pairs were known.

By modeling each trial as a series of three hemodynamic responses rather than a single response, we were able to identify changes in the pattern of activation within the time course of the task. In each session, the progression from visual cue to delay period to auditory target was associated with a progression of BOLD signal changes from occipital visual area and prefrontal areas to primarily temporal areas to temporal auditory, prefrontal, anterior cingulate, and motor regions. The hemodynamic modeling method used here cannot isolate these activations with the temporal precision that may be possible using other neuroimaging methods such as MEG (Salmelin et al., 1994; Levelt et al., 1998; Indefrey and Levelt, 2004). Nonetheless, the sequences reflect a progression of predominant relative activation increases through the task states. We do not propose that the progressions identify the stages of lexical/semantic access which occur at a far faster timescale. Instead we suggest that they reflect the change in task demands over the course of the long (~13.5 s) trials.

The task used here was extremely difficult as evinced by the large number of participants who were unable to perform the task with acceptable accuracy. Participants typically complained that the auditory stimuli were difficult to distinguish, particularly in the MRI environment. That 35% of the recruited participants were unable to perform the task in either the training room or MRI environment potentially reduces the ability to generalize the results. However, increasing task difficulty has been shown to increase BOLD response in task related areas rather than recruit additional areas in paired associate tasks (Gould et al., 2003). An easier task, while perhaps allowing the inclusion of more subjects, would thus be expected to recruit the same brain regions only to a lesser extent. Furthermore, multiple comparisons were explicitly controlled in the mixed effects analysis at the cluster level. This analysis across participants indicates that the regions identified above would be expected to be identified in the results of a random new participant. Still, the possibility that participants relied on complicated and potentially variable strategies (including linguistic strategies such as naming a pattern with a word that sounds similar to the associated distorted sound) to perform the task cannot be ruled out. The task difficulty may have biased participants to use strategies other than the purely sensory association we intended to test. The similarity of the Informed and Uninformed sessions must therefore be interpreted with caution. However, we believe that the greater BOLD signal in the rolandic operculum in the Uninformed session is evidence that participants did, in general, use a sensory strategy for all items except the Informed session animal/name items.

Previously, we reported evidence of systems level consolidation of AV memory for these stimuli in these participants (Smith et al., 2010). Contrasting recall of the AV items in the Uninformed session with the initial learning session (not reported here) showed increasing BOLD response and connectivity in lateral temporal and prefrontal regions and decreased medial temporal connectivity. The results here show these lateral temporal and prefrontal regions overlap with those used for naming. At a gross spatial scale then, our results are consistent with the hypothesis that pathways for consolidated arbitrary AV sensory memories are a subset of those underlying visual object naming. Having established this overlap, further research is necessary to better understand the nature of the overlap. Multivariate pattern analysis methods (Norman et al., 2006) may be used to determine if finer scale differences between sessions exist within the overlapping regions. Effective connectivity methods (e.g., Smith et al., 2012) are needed to fully test the alteration of the AV network interactions with the addition of linguistic processing.

## Conclusion

We presented a novel experimental paradigm to identify brain regions associated with non-linguistic, non-semantic AV memory naming using identical stimuli. The paradigm was used to disambiguate regions common to AV memory recall independent of the linguistic content of the stimuli from those specific to lexical/semantic access. We identified the left angular gyrus and middle temporal gyrus as the most likely regions indicative of activation of the lexical/semantic system that are distinct from arbitrary AV memory. We observed substantial overlap between the regions involved in both linguistic and non-linguistic versions of the task and demonstrated that linguistic regions become functionally connected to regions also active in non-linguistic AV memory. This may indicate that linguistic AV memory is a primarily a restructuring of the non-linguistic AV memory network rather than a distinct spatial pattern. Further investigation with greater spatial and temporal resolution as well as more extensive connectivity analysis is necessary to test this hypothesis.

### Conflict of interest statement

The authors declare that the research was conducted in the absence of any commercial or financial relationships that could be construed as a potential conflict of interest.
